# Endoscopic Management of Malignancy-Related Gastrointestinal Bleeding: A Comprehensive Narrative Review

**DOI:** 10.3390/medsci14010069

**Published:** 2026-02-03

**Authors:** Daniele Salvi, Maria Parmigiani, Cristiano Spada, Nicola Olivari, Stefania Piccirelli, Tommaso Schepis, Rossella Maresca, Silvia Pecere, Federico Barbaro, Paola Cesaro

**Affiliations:** 1 Department of Gastroenterology and Endoscopy, Fondazione Poliambulanza Istituto Ospedaliero, 25124 Brescia, Italy; daniele.salvi@poliambulanza.it (D.S.); maria.parmigiani@poliambulanza.it (M.P.); nicola.olivari@poliambulanza.it (N.O.); stefania.piccirelli@poliambulanza.it (S.P.); paola.cesaro@poliambulanza.it (P.C.); 2 Center for Endoscopic Research Therapeutics and Training (CERTT), Università Cattolica del Sacro Cuore, 00168 Rome, Italy; tommaso.schepis@guest.policlinicogemelli.it (T.S.); rossella.maresca12@gmail.com (R.M.); silvia.pecere@policlinicogemelli.it (S.P.); federico.barbaro@policlinicogemelli.it (F.B.); 3 Digestive Endoscopy Unit, Fondazione Policlinico Universitario Agostino Gemelli IRCCS, 00168 Rome, Italy

**Keywords:** malignant gastrointestinal bleeding, tumor-related bleeding, palliative endoscopy, multidisciplinary management, endoscopic hemostasis, topical agents, calcium electroporation

## Abstract

Malignancy-related gastrointestinal bleeding (GIB) remains a significant clinical challenge, contributing substantially to morbidity, mortality, and healthcare utilization in patients with cancer. Up to 10% of individuals with advanced malignancies develop GIB during their disease, and these episodes are frequently characterized by a high risk of rebleeding and poor long-term hemostatic control. Tumor-associated bleeding typically arises from friable, infiltrative, and highly vascular lesions that respond suboptimally to conventional endoscopic techniques such as thermal coagulation or mechanical clipping. These limitations underscore the need for improved diagnostic accuracy and more reliable therapeutic options. Recent advances in imaging modalities, including contrast-enhanced CT studies, have enhanced the ability to localize and characterize bleeding sources in complex oncologic cases. Parallel developments in endoscopic hemostasis—such as over-the-scope clips and contact-free coagulation devices—have expanded the therapeutic armamentarium for managing malignant bleeding. Clinically, topical hemostatic powders—particularly TC-325—represent a highly effective option for achieving rapid endoscopic hemostasis, supported by the strongest comparative evidence and the highest rates of immediate bleeding control among currently available technologies. In this review, we synthesize contemporary diagnostic approaches to GIB and place particular emphasis on the evolving and emerging therapeutic strategies for malignancy-related bleeding. We also highlight innovative technologies that are reshaping clinical practice and improving management options in this challenging clinical domain.

## 1. Introduction

Gastrointestinal bleeding (GIB) is a frequently encountered medical condition that remains a major source of morbidity and mortality among patients with malignancy, with GIB occurring in up to 10% of patients with advanced cancer [1]. The mortality rate for acute upper gastrointestinal bleeding can reach 10–13% [2]. Re-bleeding is also linked to increased mortality, necessitating interventions, like surgery and transfusions, leading to extended hospital stays and higher medical costs [3,4].

Gastrointestinal-related tumor bleeding accounts for 12–15% of cases of acute GI hemorrhage [5,6]. About 3–11% of acute lower GI bleeding (LGIB) is related to colonic neoplasms, whereas upper GI tract malignancy represents 2–4% of acute upper gastrointestinal hemorrhage [7,8,9]. The clinical presentation of GIB depends on the location of the bleed, volume of blood loss, and the cause of bleeding. Distinction between upper and lower GIB depends on location relative to the ligament of Treitz. Upper GIB, originating proximal to the ligament of Treitz, manifests as hematemesis, melena, or occult fecal blood [10]. Lower GIB, originating distal to the ligament of Treitz, typically presents as fresh-red blood per rectum or melanic-appearing stool depending on the location and source of bleeding [10]. Suspected small bowel bleeding is defined as ongoing or recurrent GIB without a definite source identified on esophagogastroduodenoscopy or colonoscopy, and it presents as iron-deficiency anemia and/or a positive fecal occult blood test [11].

Computed Tomography angiography (CTA) has high sensitivity (85–90%), specificity (92%), and accuracy (94–95%) for detection and localization of overt GI bleeding [12,13]. Radiologists stratify the indication to perform CTA before endoscopy based on the bleeding source: if the patient has an overt LGIB, CTA should be the first diagnostic study if the patient is hemodynamically unstable or has stable hemodynamics with a high suspicion of active bleeding. For Nonvariceal UGIB, CTA can be considered if the patient is not thought to be suitable for EGD, if there is no emergency gastroenterology coverage, or if EGD is unable to identify the site of bleeding or unable to treat the bleeding [14].

GI bleeding secondary to malignancy is a complex and challenging problem. Various modalities, such as radiotherapy, angiographic embolization, and surgery, are options for patients with malignancy-related bleeding not eligible for or after failure of endoscopic therapy. Transcatheter arterial embolization and radiotherapy are commonly employed in cases of persistent or recurrent bleeding, particularly in palliative settings, and treatment outcomes may be influenced by tumor vascularity, underlying coagulopathy, and hematological abnormalities. In addition, anticancer therapies—including antiangiogenic agents—and cancer-related thrombocytopenia further distinguish malignant from non-malignant bleeding. A multidisciplinary approach with the involvement of surgeons, radiation oncologists, interventional radiologists and gastroenterologists is required in the management of malignancy-related GI bleeding because of a lack of a protocolized approach.

Patients who benefit most from endoscopic intervention for malignant gastrointestinal bleeding are those with active bleeding, hemodynamic instability, or high-risk endoscopic findings such as active hemorrhage, nonbleeding visible vessel, or adherent clot [15,16,17,18]. Furthermore, patients presenting with GIB malignancy as the initial symptom, particularly those with localized disease or without widespread metastases, may also benefit from endoscopic intervention, although rebleeding rates remain high and overall survival is not improved [3,18]. Urgent endoscopy is indicated for patients with unstable vital signs or ongoing hematemesis/hematochezia, while stable patients may undergo early endoscopy within 24 h; prompt intervention is associated with higher rates of diagnosis and initial control of bleeding but not necessarily reduced mortality or rebleeding [15,19,20,21]. Patients with low-risk endoscopic findings (e.g., clean-based ulcers, flat pigmented spots) or stable hemodynamics may not benefit from endoscopic intervention, and oncologic management should be prioritized after initial hemostasis [16].

In this narrative review, we highlight the available therapeutic endoscopic techniques for gastrointestinal bleeding and discuss emerging innovative approaches to manage these increasingly frequent conditions.

## 2. Materials and Methods

This article is a narrative review and was not conducted according to formal PRISMA methodology or systematic review standards. We conducted a comprehensive structured search across PubMed, Scopus, and Medline, including only English-language articles published up to the end of September 2025. Our search strategy utilized detailed search strings incorporating the following terms: “Malignant gastrointestinal bleeding”, “Tumor-related bleeding”, “Palliative endoscopy”, “Endoscopic hemostasis”, “Topical Agents”, “Self-Expandable Metallic Stents”, “Calcium electroporation”, “Radiofrequency ablation”, “Hemostatic powder”, “Purastat”, “Nexpowder”, “EndoClot”, “Ankafer Blood Stopper”, “Argon plasma coagulation”, “Cryotherapy”, “TC-325”, “Over-the-scope clip”. Furthermore, we performed a manual search of the reference lists of the selected studies and related reviews to detect any other relevant publications.

We included original research articles, both prospective and retrospective clinical series, descriptive case reports, and comparative reviews reporting extractable clinical outcomes. Studies focusing exclusively on benign lesions or lacking technique-specific outcome data were excluded. Given the narrative design, formal study quality assessment and meta-analysis were not performed; larger cohorts were weighted more heavily in interpretation, while smaller series were used to highlight emerging techniques. Accordingly, potential sources of bias—including selection and publication bias, predominance of case series, lack of randomized controlled trials, and heterogeneity in bleeding etiology and outcome definitions—were considered when interpreting the findings. Available evidence is summarized in Table S1.

## 3. Injection Therapy

Endoscopic injection therapy involves the injection of substances such as epinephrine, ethanol and cyanoacrylates directly into the bleeding vessel to induce vasoconstriction or thrombosis. Injection strategies vary depending on the agent used, with mechanisms of action that may include vasoconstriction, tamponade effects, induction of platelet aggregation, sclerosis, thrombosis, and/or tissue desiccation [22].

Those techniques for malignant gastrointestinal bleeding are less effective in bleeding management than for benign lesions due to the friable, vascular nature of tumor tissue and the diffuse bleeding often encountered. Patients with focal, accessible malignant gastrointestinal bleeding sites and stable hemodynamics benefit most from endoscopic injection techniques (such as diluted epinephrine), particularly when the bleeding is not diffuse or excessively friable. In these cases, injection therapy may provide temporary hemostasis and is most effective when combined with mechanical or thermal modalities for more durable results [18,23,24]. Injection techniques are less effective in patients with advanced, diffuse, or inaccessible tumor bleeding and do not reduce recurrent bleeding or mortality [18].

### 3.1. Epinephrine

The most used injection agent is diluted epinephrine (typically 1:10,000 to 1:20,000), which provides temporary vasoconstriction and tamponade but is rarely sufficient as monotherapy and should be combined with a second modality (mechanical or thermal) for durable hemostasis [23]. Hirao et al. were the first to describe its use as a prophylaxis of post-resection bleeding in gastric cancer [25]. Despite its wide use in common practice, evidence from the literature is scarce.

### 3.2. Sclerosant Agents

Sclerosant agents such as absolute ethanol or polidocanol have been studied in peptic ulcer bleeding, even though their use is not included in guidelines. Their use in malignant bleeding is limited and not routinely recommended due to the risk of tissue necrosis and perforation [23]. Ethanol induces tissue dehydration, vascular fixation, vasoconstriction, and thrombosis, leading to rapid hemostasis.

The first to develop the technique was Asaki. In the first study, they evaluated endoscopic hemostasis using local injection of absolute ethanol to control upper gastrointestinal bleeding and included 51 patients, of whom 23 were treated for tumor-associated bleeding from gastric carcinoma, submucosal tumors, or metastatic lesions. Initial hemostasis was achieved in 100% of tumor-related cases using local absolute ethanol injection. However, late rebleeding occurred in ~6% overall (3/51 cases), with two of these linked to malignant disease: diffuse intravascular coagulation in gastric cancer and recurrent bleeding in metastatic lymphoma. Late rebleeding risk remains higher in malignancy due to coagulopathy or tumor progression, and perforation risk is increased in infiltrated or weakened tumor-bearing tissue [26]. These limitations have restricted the widespread adoption of this technique in the treatment of gastrointestinal bleeding.

### 3.3. Cyanoacrylate

Since its introduction in 1984, the widespread use of Cyanoacrylate has demonstrated its efficacy in managing bleeding from gastric varices [27]. Despite this, complications like systemic embolism, end-organ infarction and visceral fistula had been described [28,29]. Its use in malignant GI-related bleeding is based on some minor experience, with no randomized controlled trial available.

In a case report by Wang et al., primary endoscopic hemostasis for a gastrointestinal stromal tumor (GIST) bleeding was achieved with tissue adhesive obturation of the bleeding vessel. The hemostasis was successful, and no recurrence of bleeding was observed. Six days after the procedure, the patient underwent laparoscopic partial gastrectomy [30]. Similarly, Shen et al. evaluated endoscopic treatment with N-butyl-2-cyanoacrylate in 30 patients presenting with malignant gastrointestinal bleeding affecting either the upper or lower tract. Immediate technical success was achieved at all tumor bleeding sites; however, rebleeding occurred in 17% of patients within 30 days, increasing to 20% at 60 days. One instance of vascular embolization was documented as a treatment-related complication [31].

## 4. Mechanical Therapy

Mechanical endoscopic therapies offer important hemostatic options for malignant gastrointestinal bleeding, particularly when conventional techniques are insufficient. Standard endoscopic clips provide tamponade of bleeding vessels, while the Over-The-Scope Clip (OTSC) enables deeper tissue capture and has shown high technical success in refractory tumor-related hemorrhage. Endoloops, traditionally used for preventing bleeding during polyp removal, have been applied in selected cases of bleeding from gastrointestinal stromal tumors with favorable outcomes. Additionally, self-expandable metallic stents can achieve hemostasis through tumor compression, especially in obstructive lesions. Although promising, these approaches rely largely on case reports and require further evidence to define their optimal use.

### 4.1. Clips

Endoscopic clips are mechanical devices used for tamponade of bleeding vessels in malignant gastrointestinal bleeding. Their mechanism of action is direct mechanical compression of the bleeding site, which can occlude exposed or underlying vessels and promote hemostasis. Clips are typically deployed over a visible vessel or bleeding point and may also be placed on either side of the lesion to seal the underlying artery, as recommended by the American College of Gastroenterology (ACG) [32]. Technical features of endoscopic clips include rotatability, which allows precise orientation and placement, and a range of size options to accommodate different lesion characteristics and anatomical locations. Modern clips can be rotated and opened/closed multiple times before deployment, improving accuracy and effectiveness, especially in challenging locations [33]. More recently, the Over-The-Scope Clip (OTSC; Ovesco, Tubingen, Germany) was designed for tissue approximation of fistulas and perforations and has been used in refractory non-variceal GI bleeding. Chan et al. described a case series of 9 patients in whom OTSCs were used for endoscopic control of refractory or major upper gastrointestinal bleeding from lesions. Two of them were neoplastic: a bleeding from a GI tumor in the stomach and an ulcerative carcinoma of the pancreas. Technical success, defined as hemostasis achieved with the OTSC at index endoscopy, was 100%, and the clinical effectiveness was 77.8% [34].

### 4.2. Endoloops

Disposable snares, also known as endoloops, have been primarily used as prophylaxis for post-resection bleeding in pedunculate polyps’ removal. The experience with its use in neoplastic bleeding is mainly based on case reports, especially in the treatment of GIST. However, the use of endoloops to provide hemostasis in active tumor bleeding is possible; but as tumor size increases, it becomes more difficult to place the endoloop around the tumor and control bleeding from larger vessels.

Arezzo et al. were the first to describe endoscopic treatment of active bleeding from a 3.5 cm ulcerated GIST in the gastric fundus. Initial hemostasis was successfully achieved using conventional endoscopic techniques, including epinephrine injection and hemoclip placement. Since surgery was not indicated, an endoloop was applied to manage the tumor, with a second loop placed one week later. At the 4-week follow-up, the lesion had fully healed, with no signs of residual tumor or recurrent hemorrhage [35]. In another case, preoperative management of an actively bleeding gastric fundus GIST was achieved using an endoloop placed around the tumor base. Once the device was tightened, no further bleeding occurred, and the patient subsequently underwent elective tumor resection [36]. A similar report described endoloop application for a bleeding gastric GIST in a patient with advanced disease and no indication for surgery. Hemostasis was confirmed endoscopically one week after placement, and the patient was referred to palliative care, passing away two months later without recurrent bleeding [37]. Finally, a patient with a periampullary metastatic hepatocellular carcinoma tumor was treated with two endoloops placed one month apart, resulting in tumor necrosis, detachment of the mass, and stabilization of the patient’s hemoglobin levels [38].

### 4.3. Self-Expandable Metallic Stents

Endoscopic stent placement is a minimally invasive option for palliation of malignant gastrointestinal bleeding, exclusively when bleeding is associated with obstructive tumors, by restoring the lumen and compressing the tumor to achieve hemostasis. Evidence for stent placement specifically for malignant GI bleeding is limited to case reports and small series, and most guidelines focus on its role in obstruction rather than primary hemostasis; further studies are needed to clarify its efficacy for bleeding control. Moreover, for palliative management of malignant obstruction, current ESGE guidelines recommend the use of uncovered self-expanding metal stents, which are associated with limited compressive hemostatic capability [39].

Four case reports described primary hemostatic failure in patients with ulcerated esophageal and duodenal tumors despite conventional endoscopic interventions; however, immediate cessation of bleeding was achieved following placement of fully covered self-expanding metallic stents (SEMS) [40,41,42].

YuQian et al. described a case series in which SEMS were successfully used to control bleeding in four patients with esophageal tumors. Rebleeding occurred in only one patient following removal of the initial stent after four days, and hemostasis was achieved after placement of a second stent [43]. In contrast, Lee et al. emphasized caution when using SEMS for obstruction or bleeding in patients with hepatocellular carcinoma (HCC) involving the gastroduodenal region. In their series, one patient experienced hematemesis 11 days after stent deployment, likely due to the combination of HCC’s high vascularity and coagulation abnormalities associated with underlying liver cirrhosis, which may increase bleeding risk compared with other malignancies [44].

## 5. Thermal Therapy

**Figure 1:**
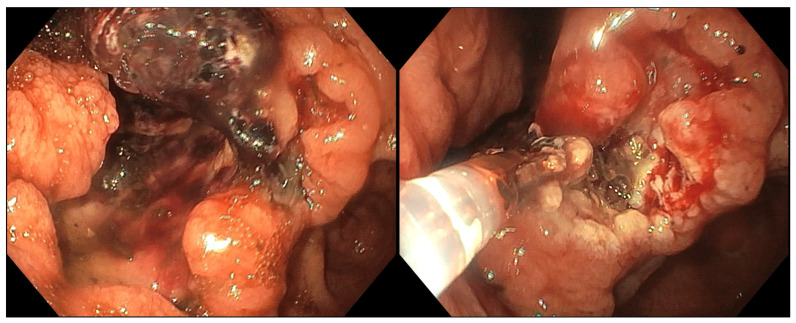
Endoscopic hemostasis of malignant gastric bleeding using a monopolar electrocautery probe in a patient with a pacemaker.

Thermal devices used in the treatment of GI bleeding include a variety of contact and noncontact modalities. Current contact thermal devices include heater probes, mono- and multipolar electrocautery probes and coagulation forceps. In Figure 1 we demonstrate an endoscopic use of a monopolar forceps for hemostasis of bleeding from a gastric neoplasm. Noncontact thermal techniques include the use of argon plasma coagulation (APC), cryoablation and laser coagulation. These methods are used to coagulate bleeding vessels or tumor surfaces.

Contact thermal devices, including bipolar electrocoagulation and heater probe, are applied directly to the bleeding site with firm pressure. The ACG recommends settings of approximately 15 W for 8–10 s for bipolar electrocoagulation and 30 J for heater probe, using a large 3.2 mm probe for optimal efficacy [32]. As demonstrated in meta-analyses of randomized controlled trials, the utilization of these techniques has been shown to result in reduced rates of further bleeding and mortality when compared with no endoscopic therapy. However, most of the data originate from non-malignant lesions [45]. The efficacy of these treatments is limited in cases of malignant bleeding due to the friable nature of tumor tissue and the tendency for diffuse bleeding.

Contact thermal therapy has become less frequently employed than non-contact therapy due to its restricted field of application, diminished initial hemostasis, and elevated rebleeding rates. Contact modalities rely on the combination of heat and selective thermocoagulation to seal a blood vessel. It is important to note that a significant proportion of tumors do not present with a solitary ulcerated vessel that can be treated. Reports of heater probes and bipolar electrocautery have demonstrated moderate-excellent initial hemostasis (67–100% initial response), yet concurrent bleeding rates of 33% to 80% have been observed [46]. Furthermore, a significant proportion of patients in these studies frequently necessitated the implementation of adjunctive interventions. Non-contact thermal modalities are used for superficial coagulation of bleeding surfaces, especially when the bleeding is diffuse or the lesion is difficult to access. Considering this evidence, the ACG and the American Gastroenterological Association recognize APC as a treatment option, though evidence quality is lower than for contact thermal devices [23,32].

### 5.1. Radiofrequency Ablation

Radiofrequency ablation (RFA) has emerged as a cornerstone therapy in management of Barrett’s esophagus and was more recently deployed for use in treatment and palliation of biliary malignancies and cystic pancreatic neoplasms, GAVE and post-attinic proctitis. RFA has been used as an effective and safe endoscopic treatment for malignant gastrointestinal bleeding. Nevertheless, most evidence comes from case series and retrospective studies, and further controlled trials are needed to establish its efficacy and optimal indications in malignant settings.

Stang et al. reported a case in which percutaneous ultrasound-guided RFA successfully controlled bleeding from a locally advanced, inoperable gastrointestinal tumor that was refractory to endoscopic therapy and unsuitable for angiographic embolization [47]. Similarly, a pilot study by Vavra et al. assessed RFA as a palliative measure for bleeding control in 12 patients with rectal cancer. In ten patients, the tumor was surgically resected immediately after ablation without local complications, while in the remaining two patients, RFA was used as the sole therapy and achieved effective hemostasis [48].

### 5.2. Argon Plasma Coagulation

Thosani et al. retrospectively analyzed endoscopic procedures performed over three consecutive years at a high-volume center to evaluate the role of APC in malignant gastrointestinal bleeding. Immediate hemostasis was achieved in all patients (10/10, 100%), with APC used alone in eight cases and combined with epinephrine injection in two. Rebleeding occurred in three patients (30%) during follow-up, and the 30-day mortality rate was 0%. Importantly, seven patients (70%) were able to continue cancer-directed therapy, including chemotherapy, radiotherapy, or both, after achieving hemostasis [49]. In contrast, Martins et al. reported no significant effect of APC on rebleeding or mortality in malignant upper GI bleeding. Their study compared 25 patients treated with APC to 28 patients who received no endoscopic intervention. Although active bleeding was more common in the APC group, baseline clinical characteristics were otherwise similar. No statistically significant differences were observed in 30-day rebleeding (33.3% vs. 14.3%, *p* = 0.104) or 30-day mortality (20.8% vs. 42.9%, *p* = 0.091) [50]. More recently, a large retrospective analysis involving 313 patients with malignant gastrointestinal bleeding found that only 22.7% underwent endoscopic therapy, with APC being the most frequently used modality. Hemostasis was achieved in 57.7% of treated cases, although APC did not appear to reduce rates of recurrent bleeding or mortality [18].

### 5.3. Cryotherapy

Endoscopic cryotherapy represents a safe, effective, and well-tolerated therapeutic option for various clinical scenarios in gastrointestinal endoscopy, including refractory Barrett’s esophagus and advanced esophageal cancer [51].

Nieto et al. reported the use of cryotherapy to manage refractory gastrointestinal bleeding in a patient with metastatic rectal cancer, achieving sustained hemostasis at 8-month follow-up after six treatment sessions [52]. In another case, persistent rectal bleeding caused by malignant invasion of the rectosigmoid colon from recurrent primary peritoneal psammocarcinoma was treated with three outpatient sessions of spray cryotherapy. The treatment was well tolerated and resulted in durable hemostasis, with improvement in hemoglobin levels and no need for hospitalization or transfusion [53]. However, potential complications should be considered: Prakash et al. described a case in which a patient developed partial large bowel obstruction due to a preexisting paraumbilical hernia following spray cryotherapy used for palliative management of rectal bleeding [54].

## 6. Topical Hemostatic Agents

The use of topical hemostatic agents has steadily increased with the development of new endoscopic tools. The ESGE recently published a technical and technology review evaluating their role in the control and prevention of gastrointestinal bleeding, reporting promising hemostatic outcomes, particularly in malignant bleeding [55]. A meta-analysis by Karna et al. examined 16 studies involving 530 patients with malignancy-related GI bleeding and demonstrated primary hemostasis in 94.1% of cases, with early and delayed rebleeding rates of 13.9% and 11.4%, respectively [56]. Although several topical hemostatic powders are discussed, the current evidence base is not equivalent: TC-325 is supported by multiple randomized controlled trials and individual patient data meta-analyses demonstrating superior immediate hemostasis. Table 1 summarizes the available evidence on the use of different hemostatic agents in malignant gastrointestinal bleeding.

### 6.1. TC-325

TC-325 (commercially available as Hemospray; Cook Medical, Winston-Salem, NC, USA) consists of an inert mineral that quickly absorbs water upon contact with blood. This absorption creates an adhesive seal, achieves mechanical tamponade, and concentrates clotting factors without directly engaging the clotting cascade.

TC-325 is the topical hemostatic agent with the strongest comparative evidence for malignant gastrointestinal bleeding, demonstrating superior immediate hemostasis and lower 30-day rebleeding rates compared to conventional endoscopic modalities. An individual patient data meta-analysis of randomized trials with 165 patients found that TC-325 achieved higher immediate hemostasis (odds ratio [OR] 46.6, 95% CI 5.89–369.1) and lower 30-day rebleeding (OR 0.28, 95% CI 0.11–0.70) than standard endoscopic therapy, with no increase in adverse events or mortality, though the certainty of evidence is low to very low due to limited sample size and event rates [57]. A multicenter randomized controlled trial confirmed these findings, reporting 100% immediate hemostasis and 2.1% 30-day rebleeding with TC-325 versus 68.6% and 21.3% for standard therapy, respectively [58]. Different meta-analyses consistently show that hemostatic powders, particularly TC-325, outperform conventional endoscopic treatments for initial hemostasis and reduce short-term rebleeding in malignancy-related GI bleeding [59,60].

More recently, Cooper et al. corroborated previous findings and additionally demonstrated that using TC-325 for hemostasis offers cost savings compared with standard endoscopic therapy, estimating a reduction of £245.88 per procedure due to fewer required interventions, shorter hospital stays, and reduced readmissions [61].

### 6.2. PuraStat

PuraStat (commercially available as Purastat; 3-D Matrix Ltd., Tokyo, Japan) is a self-assembling peptide gel developed by 3-D Matrix Ltd. with a hemostatic efficacy rate of 94% in acute GI bleeding [55]. In a study of 111 patients with acute gastrointestinal bleeding, including 15 cases of malignancy-related bleeding, PuraStat was evaluated as a primary hemostatic agent and demonstrated an overall efficacy of 94%. Most bleeding events were associated with peptic ulcers, tumors, or angiodysplasia. Success, defined as the absence of rebleeding, was observed in 91% of patients at 3 days and 87% at 7 days following initial use; in the overall cohort, success rates were 87% and 81%, respectively. Rebleeding occurred in 12% of patients within 7 days and 16% within 30 days. Among the five patients who subsequently required surgery, PuraStat provided temporary hemostasis and clinical stabilization in all cases [62].

De Nucci et al. reported a case series of 77 patients treated for acute upper and lower gastrointestinal bleeding over two years. While most bleeds were post-procedural, three patients had gastric cancer–related bleeding and two had colonic neoplasia. PuraStat was used as a salvage therapy after failure of two prior hemostatic methods, achieving initial hemostasis in 90% of cases and a rebleeding rate of 10% [63]. A recent meta-analysis of seven studies including 427 patients assessed the effectiveness of PuraStat as a hemostatic agent. The pooled rate of successful hemostasis was 93.1%, with a rebleeding rate of 8.9%. No significant differences in efficacy were observed between PuraStat used as a standalone therapy and when combined with other treatment modalities [64].

### 6.3. UI-EWD

**Figure 2:**
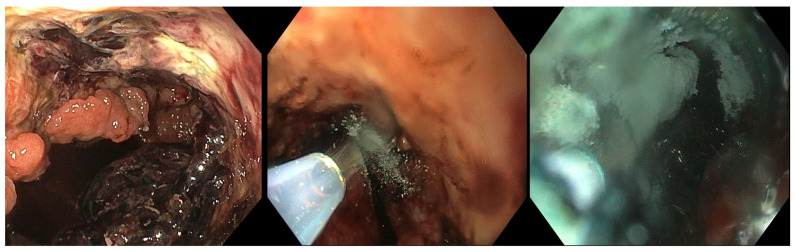
Endoscopic application of UI-EWD to control active bleeding from a malignant gastric tumor.

Upper intraluminal endoscopic wound dressing (UI-EWD) (commercially available as Nexpowder; Medtronic, Minneapolis, MN, USA) is a biocompatible natural polymer of aldehyde dextran and succinic acid-modified ε-poly. Upon contact with moisture, these two materials immediately convert into an adhesive hydrogel, creating a mechanical barrier to promote hemostasis. In Figure 2 we present the endoscopic application of UI-EWD for the management of bleeding from a gastric neoplasm.

Park et al. were the first to evaluate UI-EWD in a pilot study involving 17 patients with refractory upper gastrointestinal bleeding who had failed conventional endoscopic therapy, including four cases of neoplasm-related bleeding. UI-EWD was used as rescue therapy, achieving initial hemostasis in 16 of 17 patients (94%). The 30-day rebleeding rate was 19% (3/16), and on second-look endoscopy 24 h later, the agent remained at the treatment site in 11 of 16 patients (69%) [65].

A retrospective study of 56 patients who received UI-EWD as monotherapy for nonvariceal upper gastrointestinal bleeding reported a primary hemostasis success rate of 96.4%, with a 30-day rebleeding rate of 3.7% (2/54) [66]. Shin et al. evaluated 41 consecutive patients with upper GI tumor bleeding—including adenocarcinoma, squamous cell carcinoma, gastrointestinal stromal tumor, or lymphoma—where UI-EWD was applied either as rescue therapy after failure of conventional hemostatic modalities or as monotherapy. Overall, primary hemostasis was achieved in 97.5% of patients, with rebleeding within 28 days occurring in 22.5%. In the subgroup treated with UI-EWD as monotherapy, primary hemostasis was successful in all 23 patients, with a 28-day rebleeding rate of 26.1% [67].

Evidence for lower gastrointestinal bleeding is mainly drawn from a retrospective cohort of 55 patients who received UI-EWD either as monotherapy (n = 17) or as salvage therapy after prior endoscopic hemostasis (n = 37). In the monotherapy group, most bleeds were post-procedural or tumor-related (88.2%), and 94.1% involved lesions larger than 1 cm. Compared with a historical cohort of 112 patients treated with conventional endoscopic hemostasis for acute lower GI bleeding, UI-EWD achieved significantly higher hemostasis rates for lesions at the hepatic flexure (7.3% vs. 0%; *p* = 0.01) and for lesions larger than 4 cm (25.5% vs. 8.0%; *p* = 0.002). The cumulative 28-day rebleeding rate was 5.5% in the UI-EWD group versus 17.0% in the conventional therapy group (*p* = 0.04) [67,68].

### 6.4. EndoClot

EndoClot Polysaccharide Hemostatic System (commercially available as EndoClot PHS; Olympus, Tokyo, Japan) is a single-use endoscopic hemostatic system.

Recent evidence on the use of EndoClot for malignant gastrointestinal bleeding comes primarily from a retrospective observational study in Mexico, which included 54 patients with upper GI malignancy-related bleeding treated between 2018 and 2023. The majority received EndoClot (96%), while a smaller proportion received Hemospray (3.7%). Hemostatic powders were applied as primary therapy in 44 patients (81.5%) and as rescue therapy in combination with other modalities in 18.5% of cases. Initial hemostasis was achieved in all patients, independent of the powder type used. Rebleeding occurred in 12 patients (22.2%) within 7 days, with a cumulative 30-day rebleeding rate of 44.4%. No significant differences were observed between those treated with powder monotherapy and those who received combination therapy with argon plasma coagulation (6 vs. 3 patients; *p* = 0.146). Importantly, most patients experiencing rebleeding (81.5%) did not require additional endoscopic interventions [69].

### 6.5. Ankaferd Blood Stopper

Ankaferd Blood stopper (commercially available as ABS; Ankaferd Health Products Ltd., Istanbul, Turkey) is a novel hemostatic agent that is composed of a standardized mixture of the plants Thymys vulgaris, Glycyrrhiza glabra, Vitis vinifera, Alpinia officinarum, and Urtica dioicia. Kurt et al. retrospectively evaluated ABS in 10 patients with malignant GI bleeding, achieving hemostasis in all cases [70]. Reported primary hemostasis rates for ABS have ranged from 86% to 100%, with rebleeding rates up to 3%. These results are comparable to outcomes reported for other topical hemostatic agents, as summarized in a meta-analysis of 59 studies by Alali et al. [57].

## 7. Calcium Electroporation

Calcium electroporation (Ca-EP) represents an emerging modality for the management of neoplastic gastrointestinal bleeding. The EndoVE system, specifically developed for endoscopic Ca-EP, delivers controlled electrical pulses to increase cell membrane permeability, thereby facilitating calcium influx into tumor cells [71]. The resultant intracellular calcium overload induces apoptosis and promotes a local immune-mediated response that contributes to tumor ablation. This novel technique offers a biologically targeted and minimally invasive palliative option, although evidence remains limited and further studies are needed to define its role in malignant GI bleeding.

Bonura et al. recently reported a retrospective series of five consecutive patients with end-stage bleeding gastric cancer treated with Ca-EP. Clinical success, defined by hemoglobin stabilization or reduced transfusion requirements, was achieved in four out of five patients (80%), while one patient required additional hemostatic radiotherapy. No major or minor adverse events were observed, and all patients were discharged within 24 h. Three patients underwent repeat Ca-EP sessions due to recurrent bleeding [72].

Similarly, Adeyeye et al. described the palliative use of electroporation in 16 frail patients (median age 84.5 years) with inoperable colorectal cancer. Among 36 treatment sessions, rectal bleeding was the most frequent presenting symptom (75%). Nine patients had metastatic disease, and three had previously failed conventional therapies. Symptomatic improvement and enhanced quality of life were reported in 86.7% of patients, with transfusion or iron infusion requirements reduced by 91.7%. Median cancer-specific survival was 10 months, and no device-related complications were noted [73].

## 8. Conclusions

Malignant gastrointestinal bleeding remains a challenging clinical entity associated with substantial morbidity, high rebleeding rates, and significant healthcare utilization. Despite advances in diagnostic imaging and supportive care, endoscopic hemostasis continues to play a central role, particularly for patients with active bleeding, hemodynamic instability, or high-risk endoscopic features. Traditional modalities—including injection, mechanical, and thermal therapies—can provide temporary control but are limited by the friable, vascular nature of tumor tissue and the diffuse bleeding patterns characteristic of malignancy. The emergence of topical hemostatic powders, self-assembling peptide gels, and biopolymer-based dressings has markedly expanded therapeutic options, with accumulating evidence demonstrating high rates of immediate hemostasis and favorable safety profiles; notably, topical powders should be considered early in cases of refractory or diffuse malignant bleeding where conventional techniques are unlikely to be durable. Novel technologies such as calcium electroporation and radiofrequency ablation offer promising biologically targeted or ablative approaches, although data remain limited to early clinical studies.

In the absence of standardized treatment algorithms, management should follow a stepwise, multidisciplinary escalation strategy integrating endoscopy, interventional radiology, radiation therapy, and oncology, particularly for patients with recurrent bleeding or limited endoscopic durability. Continued prospective research and comparative studies are essential to clarify the long-term efficacy of emerging modalities, define patient selection criteria, and develop evidence-based pathways for the management of malignancy-related gastrointestinal bleeding.

## Figures and Tables

**Table 1:** Hemostatic Agents: Evidence in Upper and Lower Malignant Gastrointestinal Bleeding.

Hemostatic Agent	Evidence in Upper GI Bleeding	Evidence in Lower GI Bleeding
TC-325	+	+
PuraStat	±	±
UI-EWD	±	±
EndoClot	±	−
Ankaferd Blood Stopper	±	±

GI: Gastrointestinal; + = Randomized control trial supporting efficacy; ± = Limited or emerging clinical evidence; − = No clinical evidence currently available.

## Data Availability

No new data were created or analyzed in this study. Data sharing is not applicable to this article.

## Supplementary Materials

The following supporting information can be downloaded at: https://www.mdpi.com/article/10.3390/medsci14010069/s1, Table S1: Summary of Available Evidence for Endoscopic Techniques in the Management of Malignancy-Related Gastrointestinal Bleeding.
