# Supplementary material for: Endoscopic Management of Malignancy-Related Gastrointestinal Bleeding: A Comprehensive Narrative Review
Source: Med Sci (Basel). 2026 Feb 3;14(1):69. doi: 10.3390/medsci14010069 (PMC12921756; doi:10.3390/medsci14010069)
Supplement: Supplementary file 1 [file medsci-14-00069-s001.zip › medsci-4089385-supplementary.pdf]

Table S1. Summary of Available Evidence for Endoscopic Techniques in the Management of Malignancy-Related Gastrointestinal Bleeding

Author (year)	Design	Technique	GI-tract location	Patients N	Outcome
Shen et al. (2024)	Retrospective study	Cyanoacrylate	Upper and lower GIST	30	100% Immediate hemostasis<br>17% rebleeding at 30 days<br>20% at 60 days
Wang et al. (2021)	Case report	Cyanoacrylate	Gastric GIST	1	100% Immediate hemostasis<br>0% rebleeding
Chan et al. (2014)	Case series	OTSC	Upper GIST, pancreatic carcinoma with duodenal invasion	2	100% Immediate hemostasis<br>50% rebleeding
Kashani et al. (2015)	Case report	Endoloops	Metastatic periampullary HCC	1	100% Immediate hemostasis
Brkic et al. (2009)	Case report	Endoloops	Gastric GIST	1	100% Immediate hemostasis
Arezzo et al. (2011)	Case report	Endoloops	Gastric GIST	1	100% Immediate hemostasis
Retes et al. (2015)	Case report	Endoloops	Gastric GIST	1	100% Immediate hemostasis
Yen et al. (2015)	Case report	SEMS	Duodenal carcinoma	1	100% Immediate hemostasis
Orii et al. (2016)	Case report	SEMS	Duodenal carcinoma	1	100% Immediate hemostasis
Bilal et al. (2021)	Case report	SEMS	Esophageal carcinoma	1	100% Immediate hemostasis
YuQian et al. (2014)	Case series	SEMS	Esophageal carcinoma	4	100% Immediate hemostasis<br>1 rebleeding after removal
Lee et al. (2012)	Case series	SEMS	Advanced HCC with GI involvement	7	100% Immediate hemostasis.<br>14% rebleeding
Stang et al. (2004)	Case report	RFA	Gastric carcinoma	1	100% Immediate hemostasis
Vavra et al. (2009)	Case series	RFA	Rectosigmoid carcinoma	12	100% Immediate hemostasis
Thosani et al. (2014)	Retrospective study	Argon Plasma Coagulation	Primary and metastatic GI tumors	10	100% Immediate hemostasis<br>30% rebleeding
Martins et al. (2016)	Retrospective study	Argon Plasma Coagulation	Primary and metastatic upper GI tumors	25	73.3% Immediate hemostasis<br>33.3% rebleeding

Abu-Sbeih et al. (2022)	Retrospective cohort study	Argon Plasma Coagulation	Primary and metastatic GI tumors	45	57.7% Immediate hemostasis<br>39% rebleeding
Nieto et al. (2016)	Case report	Cryotherapy	Rectal cancer	1	100% Immediate hemostasis
Prakash et al. (2017)	Case report	Cryotherapy	Rectal cancer	1	100% Immediate hemostasis
Alp et al. (2025)	Case report	Cryotherapy	Colorectal cancer	1	100% Immediate hemostasis
Chen et al. (2020)	Randomized control trial	TC-325	Upper GI tumors	10*	90% Immediate hemostasis<br>20% rebleeding at 30 days
Pittayanon et al. (2023)	Randomized control trial	TC-325	Upper and lower GI tumors	55*	100% Immediate hemostasis<br>2.1% rebleeding at 30 days
de Nucci et al. (2020)	Retrospective study	PuraStat	Gastric and colonic carcinoma	5	100% Immediate hemostasis<br>60% rebleeding
Branchi et al. (2022)	Prospective study	PuraStat	GI-tumor	15	88% Immediate hemostasis<br>16% rebleeding
Park et al. (2019)	Retrospective study	UI-EWD	Upper GI carcinoma	1	100% Immediate hemostasis<br>No rebleeding
Park et al. (2019)	Prospective study	UI-EWD	Primary upper GI cancer, lymphoma, metastatic cancer	4	100% Immediate hemostasis
Shin et al. (2021)	Retrospective study	UI-EWD	Primary upper GI cancer, lymphoma, metastatic cancer	41	97.5% Immediate hemostasis<br>22.5% rebleeding at 28 days
Cha et al. (2022)	Retrospective study	UI-EWD	Colorectal cancer	5	100% Immediate hemostasis<br>No rebleeding at 28 days
Pérez-Macías et al. (2025)	Retrospective study	EndoClot	Primary and metastatic upper GI tumors	54	100% Immediate hemostasis<br>22.2% rebleeding at 7 days<br>44.4% at 30 days
Kurt et al. (2010)	Case series	Ankaferd Blood Stopper	Gastric and rectal cancers	10	100% Immediate hemostasis
Bonura et al. (2025)	Case series	Calcium-Electroporation	Gastric cancer	5	100% Immediate hemostasis<br>60% rebleeding
Adeyeye et al. (2025)	Case series	Calcium-Electroporation	Colorectal cancer	16	75% Immediate hemostasis

*Intervention arm; GIST: Gastrointestinal stromal tumor; OTSC: Over-the-scope clips; RFA: Radiofrequency ablation; HCC: Hepatocellular carcinoma; GI: gastrointestinal; UI-EWD: Upper intraluminal endoscopic wound dressing
